# Synthesis, Antiproliferative Effect and In Silico LogP Prediction of BIM-23052 Analogs Containing Tyr Instead of Phe

**DOI:** 10.3390/pharmaceutics15041123

**Published:** 2023-03-31

**Authors:** Dancho Danalev, Ivan Iliev, Stefan Dobrev, Silvia Angelova, Stoiko Petrin, Tatyana Dzimbova, Elena Ivanova, Dessislava Borisova, Emilia Naydenova

**Affiliations:** 1 Biotechnology Department, University of Chemical Technology and Metallurgy, 8 Kliment Ohridski Blvd., 1756 Sofia, Bulgaria; stpetrin@uctm.edu; 2 Institute of Experimental Morphology, Pathology and Anthropology with Museum, Bulgarian Academy of Sciences, Acad. G. Bonchev Str., Bl. 25, 1113 Sofia, Bulgaria; taparsky@abv.bg (I.I.); elena9512@abv.bg (E.I.); 3 Institute of Optical Materials and Technologies “Acad. J. Malinowski”, Bulgarian Academy of Sciences, Acad. G. Bonchev Str., Bl. 109, 1113 Sofia, Bulgaria; sdobrev@iom.bas.bg (S.D.); sea@iomt.bas.bg (S.A.); 4 Department Sport, Faculty of Public Health, Health Care and Sport, South-West University “Neofit Rilski”, 2700 Blagoevgrad, Bulgaria; tania_dzimbova@abv.bg; 5 Organic Chemistry Department, University of Chemical Technology and Metallurgy, 8 Kliment Ohridski Blvd., 1756 Sofia, Bulgaria; dborissova@yahoo.com (D.B.); emilia@uctm.edu (E.N.)

**Keywords:** BIM-23052 analogs, somatostatin, lipophilicity, antiproliferative effect, cytotoxicity

## Abstract

(1) Background: Hydrophobicity (or lipophilicity) is a limiting factor in the ability of molecules to pass through cell membranes and to perform their function. The ability to efficiently access cytosol is especially important when a synthetic compound has the potential to become a drug substance. D-Phe-Phe-Phe-D-Trp-Lys-Thr-Phe-Thr-NH_2_ (BIM-23052) is a linear analog of somatostatin with established in vitro GH-inhibitory activity in nanomolar (nm) concentrations and high affinity to different somatostatin receptors. (2) Methods: Series of analogs of BIM-23052 were synthesized where Phe residue(s) in the BIM-23052 molecule were replaced with Tyr using standard SPPS, Fmoc/t-Bu strategy. Analyses of target compounds were performed using HPLC/MS technique. Toxicity and antiproliferative activity were studied using in vitro NRU and MTT assays. The values of logP (partition coefficient in octanol/water) for BIM-23052 and its analogs were calculated. (3) Results: The obtained data show the best antiproliferative effect against studied cancer cells for compound D-Phe-Phe-Phe-D-Trp-Lys-Thr-Tyr^7^-Thr-NH_2_ (DD8), the most lipophilic compound according to the predicted logP values. (4) Conclusions: Multiple analyses of the obtained data reveal that compound D-Phe-Phe-Phe-D-Trp-Lys-Thr-Tyr^7^-Thr-NH_2_ (DD8) where one Phe is replaced by Tyr has the best combination of cytotoxicity, antiproliferative effect and hydrolytic stability.

## 1. Introduction

Somatostatin (SST) is a cyclic tetradecapeptide that exerts diverse activities, including strong regulatory effects throughout the body. Binding specific G-protein-bounded somatostatin receptors SST inhibits secretion of many hormones as well as tumor cell growth [1]. SST is a naturally occurring hormone secreted mainly in the nervous and digestive systems. It handles the normal release of hormones but is unable to cope with neuroendocrine cancer syndromes. The properties of SST are carried out via a family of five G protein-coupled receptors named SSTR 1 to 5 [2,3,4,5,6]. The latter are differently distributed not only in many places in the human body but also in some tumor cells. It was found that these receptors are overexpressed on the surface of many tumor cells, mostly those that arise in the lung, brain, digestive pancreatic tract, prostate, lymphatic system, etc. [7,8,9,10,11,12,13]. The most common SST analog drugs used are Lanreotide and Octreotide; they are clinically used in patients with locally advanced or metastatic gastroenteropancreatic neuroendocrine tumors (islet cell tumors). These medicines bind to the SST receptors and help them to both decrease hormone secretion and growth.

D-Phe-Phe-Phe-D-Trp-Lys-Thr-Phe-Thr-NH_2_ (BIM-23052), a linear analog of SST, has established vitro GH-inhibitory activity at low concentrations (nm range) and high affinity to several SST receptors (SSTRs) [14]. Many studies on this structure have revealed different structure–activity relationships. Studying SST-14, Viber et al. revealed that the tetrapeptide sequence Phe-Trp-Lys-Thr from the somatostatin molecule is responsible for specific β-folding and plays a key role in SST activity [15]. Further studies show that Trp and Lys are required for activity, while Phe and Thr can be replaced by Tyr, Ser, or Val. Addition of D-Phe at the N-terminus and L-Thr at the C-terminus and substitution of L-Trp with a D-form at position 8 all reduce the degradation of the resulting analogs [16]. Based on these modifications, several somatostatin analogs, Octreotide, Vapreotide, Lanreotide, and Seglitide, have been created and have been found to have wide application in medical practice [17,18,19,20]. In addition, Staykova et al. in several studies replaced some natural amino acids from the BIM-23052 structure with unnatural amino acids and found that some modifications exert a positive effect on the antiproliferative properties of newly synthesized peptides [21,22,23].

Searching for new structured analogs of SST with better pharmacodynamics and pharmacokinetics has continued in present years [24,25]. Our previous investigations showed that incorporation of α,α-dialkylated amino acids significantly affected the in vitro antitumor activity of the modified BIM-23052 analogs [26]. Additionally, several new analogs of BIM-23052 containing halogenated Phe were synthesized and studied by our group. This investigation revealed that the analog with one Phe(4-F) residue in the primary structure has good antiproliferative activity combined with acceptable cytotoxicity [27].

**Figure 1:**
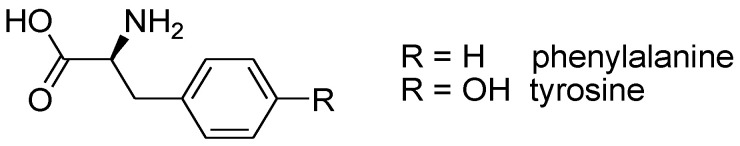
Chemical structure of Phe and Tyr aromatic amino acids.

Tyrosine (Tyr) is a structural analog of phenylalanine (Phe), containing an additional OH group in the structure (Figure 1).

Moreover, both amino acids have the same metabolic pathway for their synthesis. However, the seemingly simple replacement of Phe with Tyr in the primary structure of peptides undoubtedly changes the hydrophobicity/lipophilicity of the molecule. The hydroxy function could play an additional role in the process of bonding to specific receptors or enzymes. Lipophilicity is an important descriptor that helps scientists to predict and understand the transport and impact of chemicals in physiological systems. For example, lipophilicity is an essential property of the drug and a key factor for cell penetration ability and plays a crucial role for bioavailability of the drug’s active constituent(s) in the body. It is usually expressed as logP, which represents the ratio of the solubility of a compound in *n*-octanol to the aqueous medium. LogP values are indispensable for many industries and areas of research (especially to the pharmaceutical/biotech ones) in determining how to deliver chemical substances to specific sites or to eliminate chemicals from others [28]. It is generally accepted that drug molecules must be lipophilic in order to be well absorbed. According to Lipinski’s rule of five (Ro5), the calculated logP value should be <5 for compounds intended for oral administration [29]. Herewith, we endeavor to continue our previous work on somatostatin linear analogs and to investigate the structure–property relationship of structures that have undergone structural modifications (SST analogs) and to identify those changes that lead to increased biological activity. The strategy chosen was used to replace Phe residue(s) in the BIM-23052 molecule with Tyr. The cytotoxicity and antiproliferative properties of the newly synthesized analogs against different commonly used cell lines were studied. In silico logP prediction of BIM-23052 analogs containing Tyr instead of Phe was used to assess whether the lipophilicity of the compounds falls within the so-called “therapeutically relevant pharmacokinetic space”. In addition, in the N-terminus, both L- and D-Tyr were used in order to evaluate the role of amino acid configuration on the biological properties and lipophilicity of the molecule.

## 2. Materials and Methods

### 2.1. Materials

The solid phase carrier Fmoc-Rink Amide MBHA resin, the specifically protected amino acids needed for target structure synthesis Fmoc-L/D-Phe-OH, Fmoc-L/D-Tyr(OBu^t^)-OH, Fmoc-Thr(OBu^t^)-OH, Fmoc-Lys(Boc)-OH and Fmoc-D-Trp(Boc)-OH, activation agents N,N,N’,N’-tetramethyl-O-(1H-benzotriazol-1-yl)uronium hexafluorophosphate (HBTU) and N,N’-Diisopropylcarbodiimide (DIC), trifluoroacetic acid (TFA), scavenger triisopropylsilane (TIS) and base N,N-Diisopropylethylamine (DIPEA) were purchased from Iris Biotech (Wunsiedel, Germany). The solvents N,N’-dimetylformamide (DMF) and dichloromethane (DCM) were obtained from Valerus (Sofia, Bulgaria), and 4-N,N-dimethylaminopyridine (DMAP) was obtained from Sigma-Aldrich (Ansbach, Germany). All reagents and solvents were used as purchased without any additional purification or pretreatment.

### 2.2. Peptide Synthesis and Chemical Analysis

The aimed peptides were synthesized using the conventional solid-phase peptide synthesis (SPPS), Fmoc (9-fluorenylmethoxycarbonyl)/OBu^t^ strategy. Rink-amide MBHA resins were used as a resin in order to obtain the aimed C-terminal amides. HBTU or DIC were used as condensation reagents, with DIPEA as a base or DMAP as catalysts, depending on the condensation agent. The following molar ratios of reagents were used for the realization of coupling reactions:

- amino acid/HBTU/DIPEA/resin 3/3/9/1;
- amino acid/DIC/resin 3/3/1 with a catalytic amount of DMAP.

The ^α^N-Fmoc-protecting group was removed during every step by treatment with 20% piperidine solution in DMF. Both deprotection and condensation reactions were monitored by Kaiser test. The cleavage of aimed peptides from the resin was performed, using a mixture of 95% TFA, 2.5% TIS and 2.5% dH_2_O. The obtained as oils in TFA peptides were further precipitated in cold dry diethyl ether. Liquid chromatography on Shimadzu LC MS/MS 8045 apparatus was used to monitored peptide purity in the following conditions:

A binary linear gradient with phase A: H_2_O (10% AcCN; 0.1% HCOOH) and phase B: AcCN (5 % H_2_O; 0,1 % HCOOH):
Time (min)0.0110.0015.0015.5022.00m.ph. А (%)80558080m.ph. B (%)2095952020

The other parameters of the chromatographic system were:

- a column Agilent Poroshell 120, 100 × 4.6 mm;
- elution flow: 0.30 mL/min;
- temperature of column 40 °C.

The ESI+ MS in SCAN mode was used to prove structures of newly synthesized peptides in the following conditions: nebulizing gas flow 3 L/min, heating gas flow 10 L/min, interface temperature 350 °C, DL temperature 200 °C, heat block temperature 400 and drying gas flow 10 L/min.

The optical rotation was measured at c = 1 in methanol. Melting points of the target compound were measured on melting point meter M3000 by A. KRÜSS Optronic GmbH. All data and constants for the synthesized aimed peptides are presented in Table 1 and Table 2.

### 2.3. Cell Lines

The breast cancer cells MDA-MB-231 (ATCC: HTB-26) and MCF-7 (ATCC: HTB-22) were used to determine antiproliferative activity under in vitro conditions. The breast epithelial cells (MCF-10A, ATCC: CRL-10317) and mouse embryonic fibroblasts (BALB/c 3T3 clone A31, ATCC: CCL-163) were used as control. Cell cultures were purchased from American Type Cultures Collection (ATCC, Manassas, Virginia, USA). In cell culture, growth medium DMEM-high glucose, 10% (*v*/*v*) FBS and antibiotics (Sigma-Aldrich, Schnelldorf, Germany) were used. Cells were incubated at 37 °C, 5% CO_2_ and 95% humidity. Cells were grown in plastic flasks with a surface of 75 cm^2^ (Deltalab S.L., Barcelona, Spain).

### 2.4. Safety Test (3T3 NRU Assay)

A safety test was performed on the method BALB/c 3T3 Neutral Red Uptake cytotoxicity Assay [30,31]. The data generated from the in vitro cytotoxicity assay can be used to determine the safety level of the investigated substances and can predict the starting doses for in vivo toxicity assays. The cells (BALB/c 3T3, clone A31) were seeded at 1 × 10^4^ cells/100 μL/well in 96-well flat-bottomed microplates. The plates were placed in a thermostat at 37 °C, 5% CO_2_ and 95% humidity for 24 h. Peptides were pre-dissolved in DMSO and then culture medium (DMEM) was added. The cells were treated with the BIM-23052 analogs at concentrations from 15 to 4000 mm. The treated cell culture was incubated for 24 h in a thermostat under standard conditions. The microplates were then washed twice with PBS, and Neutral Red Desorb (1% acetic acid/50% ethanol/49% water) was added. Absorbance was measured by a microplate reader at a wavelength of 540 nm.

### 2.5. Cell Viability (MTT-Assay)

Cell viability was determined by a colorimetric MTT-dye reduction assay [32]. The method is based on an enzyme reaction leading to the reduction of 2,5-diphenyl-2H-tetrazolium bromide (MTT) to formazan crystals. The concentration of formazan, which is directly proportional to cell viability, was determined by a spectrophotometric method. Cells (MCF-7, MDA-MB-231 and MCF-10A) were seeded at a 1 × 10^3^ cells/100 µL/well in 96-well microplates. The plates were placed in a thermostat under standard conditions for 24 h. Cells were then treated with the peptide analogs at concentrations ranging from 7.5 to 2000 μm. The cells were then incubated in a thermostat for 72 h. The formazan concentration was measured spectrophotometrically by a microplate reader at a wavelength of 540 nm. The antiproliferative potential of BIM-23052 analogs was presented as IC_50_ values, calculated using GraphPad Prizm Software (San Diego, CA, USA).

### 2.6. In Silico LogP Prediction

The logP values of the peptides were determined using HyperChem 8.0.10 [33]. The calculations were performed for both non-pre-optimized and pre-optimized structures. Pre-optimization was performed using the semi-empirical AM1 method [34] and a small Pople double zeta basis set (3–21 G [35]) as employed in the G09 software [36]. All calculations were performed on singly charged amide forms of the peptides (the charge being on the Lys residue).

## 3. Results

### 3.1. Synthesis of Aimed Peptides

A series of peptide analogs of D-Phe-Phe-Phe-D-Trp-Lys-Thr-Phe-Thr-NH_2_ (BIM-23052) were synthesized by replacement of Phe residues with Tyr from the C- to the N- terminus in the positions 1, 2, 3 and 7 of the target molecule. All data on the newly obtained compounds are presented in Table 1.

In addition, analogs of BIM-23052, containing L-Tyr at the N-terminus, were synthesized to assess the impact of D- with L-amino acid replacement on biological activity and lipophilicity. The newly synthesized analogs were characterized, and all analytical data are presented in Table 2.

### 3.2. Cytotoxicity

**Figure 2:**
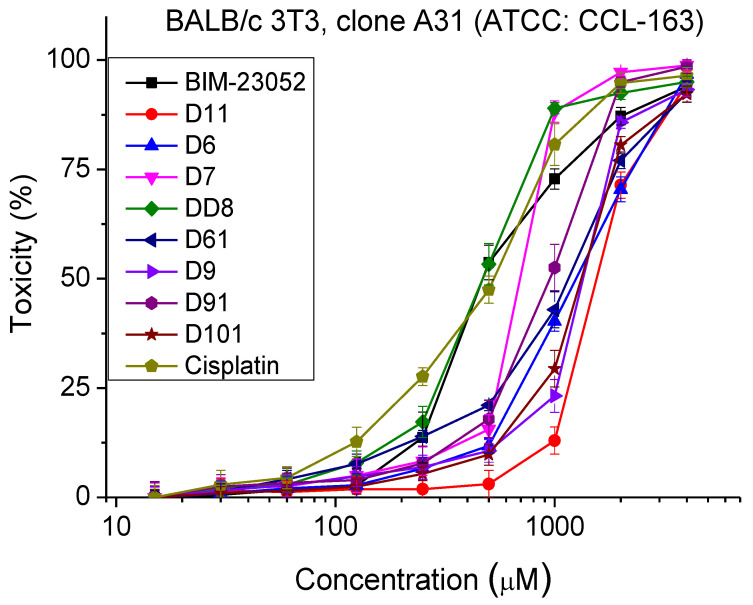
Dose–response curves for cytotoxicity of BIM-23052 analogs determined in mouse embryonic fibroblasts (BALB/c 3T3 clone A31, ATCC^®^ CCL-163™). Mean values are presented ± SD from three independent experiments, *n* = 6.

The safety test of BIM-23052 analogues was carried out by Neutral Red Uptake cytotoxicity Assay. Cell cultures were incubated with the tested peptide analogs for 24 h. The observed cytotoxic effects were of a dose-dependent type. The sigmoidal dose–response curves are presented in Figure 2. The peptide analogs tested did not exhibit toxic effects at concentrations lower than 200 µm. The calculated CC_50_ values ± SD are presented in Table 3. An analysis of the results shows that the most toxic peptide analogs were DD8 and D7, with CC_50_ = 483 ± 34 and 737 ± 54 µm, respectively. The cytotoxicity of the other investigated analogs is significantly lower (*p* < 0.001), around and above 1000 μm.

### 3.3. Antiproliferative Activity

**Figure 3:**
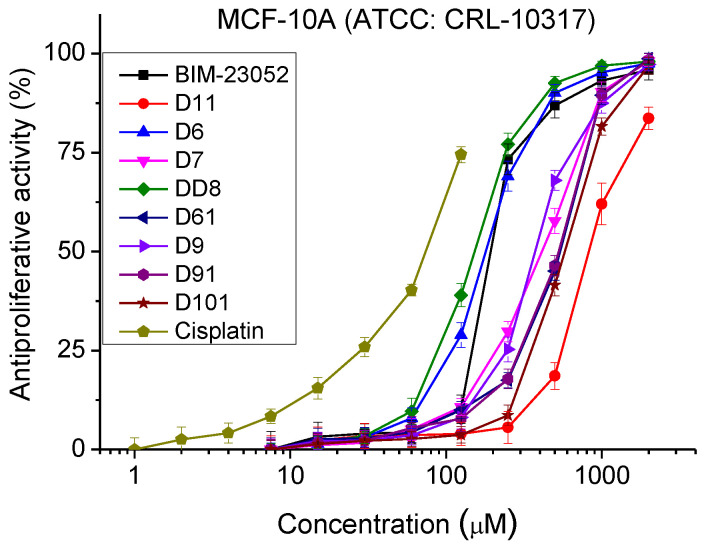

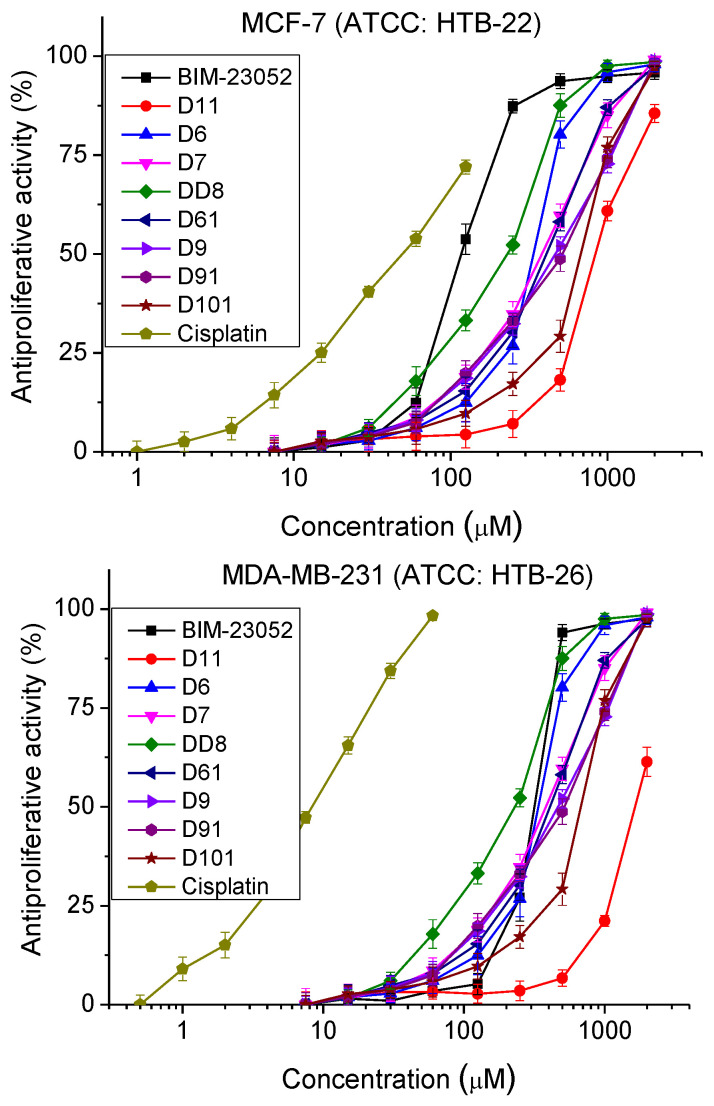
Dose–response curves for antiproliferative activity of BIM-23052 analogs determined in MCF-10A (ATCC^®^ CRL-10317^TM^), MCF-7 (ATCC^®^ HTB-22^TM^) and MDA-MB-231 (ATCC^®^ HTB-26^TM^) cell lines. Mean values are presented ± SD from three independent experiments, *n* = 6.

The antiproliferative activity of BIM-23052 analogs was studied by MTT assay. The normal and tumor cell cultures were incubated with the peptide analogs with a concentration range from 7.5 to 2000 μm for 72 h. The antiproliferative activity was determined (Figure 3 and Table 3). MCF-10A and MCF-7 cell lines showed similar sensitivity to the peptide analogs tested. In the MDA-MB-231 cell line, we observed significantly lower antiproliferative activity compared to MCF-10A and MCF-7 cells. In all cell lines, substances D6 and DD8 showed the strongest antiproliferative activity compared to the other investigated peptide analogs.

### 3.4. Selectivity

The selectivity index (SI) is calculated as a ratio between IC_50_ values of MCF-10A and IC_50_ of the corresponding tumor cell lines for each of the test substances. Low selectivity against the MDA-MB-231 tumor cell line was observed in all peptides tested (Table 4). On the MCF-7 cell line, SI values close to 1 were observed. This is probably due to a similar mechanism of inhibition of cell proliferation in the MCF-7 and MCF-10A cell lines. Peptide analog D61 showed weak selectivity in the MCF-7 cells (SI = 1.3).

### 3.5. LogP Prediction

**Figure 4:**
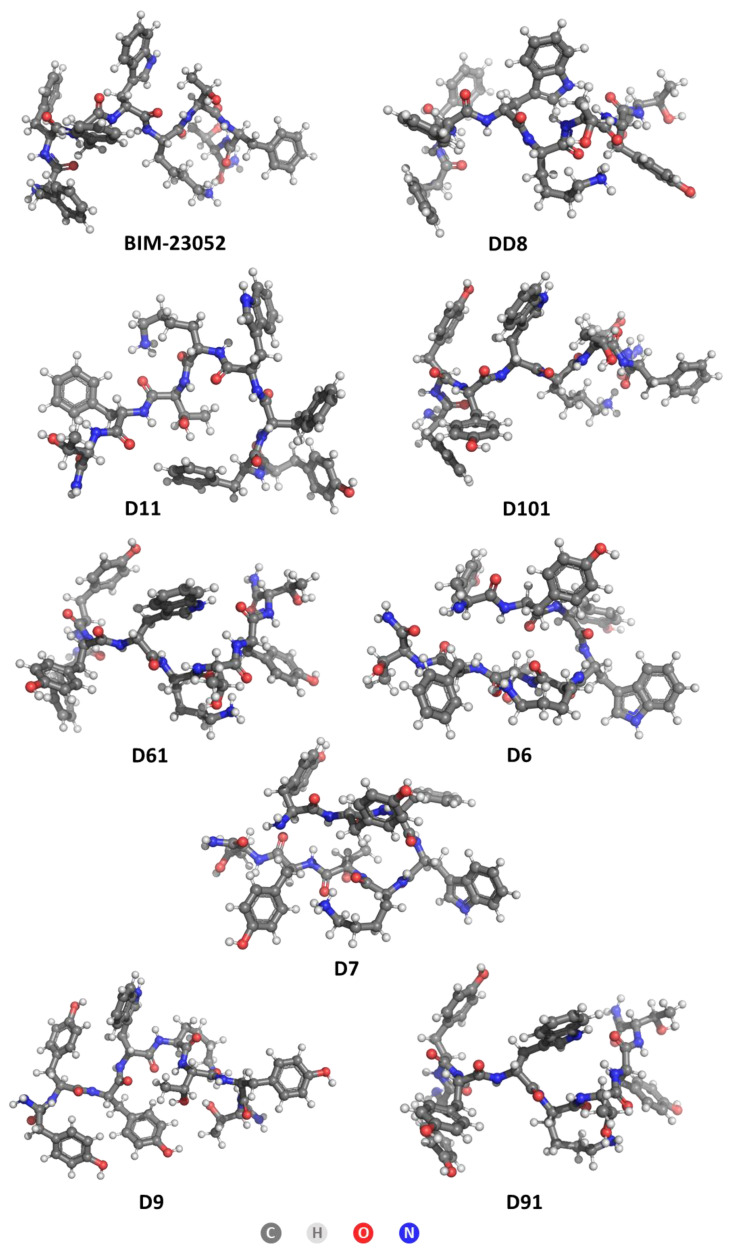
Optimized structures of BIM-23052 and its analogs.

The lipophilicity/hydrophilicity of peptides are very important characteristics for the rational design and drug discovery of bioactive molecules (and peptides). The logP calculations were performed for both non-pre-optimized and pre-optimized structures. In general, geometry optimization is a computational method used to predict the 3D (three-dimensional) arrangement of the atoms in a molecule by means of minimization of total energy, E_T_, of the system. Since geometry optimization (the process of changing the system’s geometry) is a key component of most computational chemistry studies that deal with the structure and/or reactivity of molecules, we decided to perform such calculations even though they are not typical of logP prediction calculations. The optimized structures of BIM-23052 analogs are shown in Figure 4. The calculated logP values for both non-pre-optimized structures and pre-optimized structures are given in Table 5.

### 3.6. Hydrolytic Stability

Hydrolytic stability of the target peptides was tested for 24 h in two model systems at pH values that mimic human pH in the stomach and small intestine. The used buffers were prepared according to the European Pharmacopoeia, 6th Edition:

(i) Buffer with pH 2.0–6.57 g KCl was dissolved in water (CO_2_ free) and 119.0 mL 0.1 mol/L HCl was added. The obtained solution was completed to 1000.0 mL with distilled water (dH_2_O).
(ii) Buffer with pH 9.0–1000.0 mL of solution I was mixed with 420.0 mL of solution II. Solution I: 6.18 g H_3_BO_3_ was dissolved in 0.1 mol/L KCl and was completed to 1000.0 mL with the same solvent; Solution II: 0.1 mol/L NaOH.

The chromatographic system used for determination of the hydrolytic stability included an HPLC model Perkin-Elmer series 200, USA, Agilent ZORBAX Eclipse Plus C18 column (pore size 5 μ, internal diameter 4.6 mm and length 150 mm., Agilent Technologies, USA), UV detector (PerkinElmer series200, USA) set at 275 nm, and flow rate 0.70 mL/min with isocratic elution using a mobile phase: Water:Acetonitrile 70:30 at room temperature, sample injection volume: 20 μL.

All samples were dissolved in an ultrasonic bath for 15 min and filtered through a 0.40 µm filter before injection into the chromatographic system. The obtained data at pH 9.0 for 24 h are presented in Table 6, and both those at pH 2.0 and 9.0 are presented in the Discussion section.

### 3.7. Docking Studies

Somatostatin receptors were modeled as previously was described [26]. Three-dimensional structures of the ligands were modeled in Avogadro (http://www.chemcomp.com, accessed on 1 January 2023). Ligands were protonated at physiological pH 7.4. Docking was carried out with GOLD 5.2 software. The binding sites for SSTRs, in the literature [37] are defined as residues Ser-Gln-Leu-Ser (305–308) for SSTR1, Phe-Asp-Phe-Val (294–297) for sstr2, Tyr-Phe-Leu-Val (295–297) for SSTR3, Asn-His-Val-Ser (293–296) for SSTR4, and Tyr-Phe-Phe-Val (286–289) for SSTR5. For docking we used the first residues from the sequences and the space within a 10 Å radius from them. ChemScore scoring function of GOLD was used, and the energies of the obtained ligand–receptor complexes for the most active compound DD8 were calculated using Molegro Molecular Viewer (http://molegro.com/index.php, accessed on 1 January 2023). The obtained results are summarized in Table 7.

## 4. Discussion

### 4.1. BIM-23052 Analogs Synthesis and Characterization

A standard SPPS Fmoc/OBu^t^ strategy on Rink Amide Resin as solid phase carrier was used for the synthesis of target peptides with the general formula D/L-Xxx/Yyy^1^-Xxx/Yyy^2^-Xxx/Yyy^3^-D-Trp-Lys-Thr-Xxx/Yyy^7^-Thr-NH_2_, where Xxx is Phe, and Yyy is Tyr. The first step of condensation is always performed by using amino acid/HBTU/DIPEA/resin system in a molar ratio of 3/3/9/1. If the condensation step is not completed (positive Kaiser test), a second condensation reaction is realized using amino acid/DIC/resin in a molar ratio of 3/3/1 and catalytic quantity of DMAP. In all cases, the second condensation was successful. After the final step of deprotection and removal from the resin, all target molecules were crystalized in dry cold ether and were subjected to analysis. All analytical data are summarized in Table 1 and Table 2.

### 4.2. Cytotoxicity, Docking Calculations, Antiproliferative Effect and Lipophilicity

Cytotoxicity studies have shown that the tested peptides do not show a cytotoxic effect at concentrations less than 250 µM. The cytotoxicity of BIM-23052 is similar to that of cisplatin (IC_50_ = 483.10 and 537.35 μm). The BIM-23052 analogs containing Tyr instead of Phe have significantly lower toxicity. For analogs D11, D6, D61, D9 and D101, the IC_50_ values significantly exceed 1000 μm, which is an indicator of a high level of safety. The most active compound DD8 has cytotoxicity equal to this of the parent compound BIM-23052. The logP values for this analog where only one Phe is substituted with Tyr show that the compound is lipophilic. Thus, compound DD8 could penetrate the cell membrane better than the other tested analogs, which means that additional mechanisms of action as anticancer agents are also possible for this peptide. In addition, the realized experiments revealed that subsequent substitution of Phe with Tyr residues does not lead to better antiproliferative activity. An exception is revealed for analog D6 with three Tyr (H-L-Tyr^1^-Tyr^2^-Tyr^3^-D-Trp-Lys-Thr-Phe-Thr-NH_2_). In this case, the N-terminal Tyr residue has a L-configuration. At the same time, docking calculations show weak selectivity of this analog according to the SSTRs, similar to the parent compound BIM-23052 and in contrast to DD8, where high selectivity is revealed by docking calculations according to SSTR1.

However, the newly synthesized peptides do not show any selectivity for the tumor cell lines used in the experiments. Weak selectivity for the MCF-7 cell line (luminal-A type model, hormone-dependent breast cancer) commensurate with this of the parent molecule BIM-23052, and standard Cisplatin was observed only for compound D61, in which three Phe were replaced by Tyr. The calculated logP values indicate that this compound is less lipophilic than BIM-23052 and would have more difficulty penetrating the cell membrane to induce apoptosis by entering the cell.

Tyrosine is expected to have a lower logP value than phenylalanine because it contains an additional hydroxyl group that contributes to its solubility in water. This is evident from numerous studies of the amino acid logP found in the literature [38,39,40]. However, when Tyr is incorporated in the primary structure of peptide molecules instead of Phe, the obtained results do not exactly match with this conclusion. The obtained results reveal that the addition of tyrosine residues decreases the lipophilicity by about 0.3 for each residue in the non-optimized structures, but the optimized single substitution analogs H-D-Phe-Phe-Phe-D-Trp-Lys-Thr-**Tyr**^7^-Thr-NH_2_ (DD8) and H-D-**Tyr^1^**-Phe-Phe-D-Trp-Lys-Thr-Phe-Thr-NH_2_ (D11) are actually more lipophilic than the parent structure BIM-23052. The obtained data for the cytotoxicity of DD8 (IC_50_ = 482.93 ± 33.88 µm) also match with the results from in silico calculations, and they show that this analog is the most cytotoxic one, which means that it influences the cell membrane not only specific to SSTR. However, the displacement of the Tyr residue closer to the N-terminus in D11 leads to a decrease in cytotoxicity, which clearly shows that the placement of the substituted residue plays a significant role in the penetration of the cell membrane. At the same time, analog D11 has the lowest activity between all other Tyr-containing compounds, which means that the D-Phe in the N-terminus is also key for bonding to the SSTR receptors. Peptide H-D-Phe-Tyr^2^-Tyr^3^-D-Trp-Lys-Thr-Phe-Thr-NH_2_ (D101), where two tyrosines are added next to the N-terminus of the peptide, has similar logP to BIM-23052 but a several-times lower antiproliferative effect on all tested cancer cell lines. This result reveals that Phe residues assure better selectivity to SSTRs, but this substitution does not provide an additional option for action of the peptides through membrane penetration. All optimized structures show a lipophilic nature, while the non-optimized structures vary from slightly hydrophilic (D61, D9, D91 and D6) to slightly lipophilic (BIM-23052, DD8, D101, D7, D11). It can be assumed that this series of compounds affects cancer cells only by interaction with SSTS and not through cell penetration mechanisms. Finally, these results lead to the conclusion that the hydrophobicity/lipophilicity of the molecule also depend on the secondary structure of the molecule.

The effect of L-Tyr at the N-terminus can be measured when comparing both compounds H-L-Tyr^1^-Tyr^2^-Tyr^3^-D-Trp-Lys-Thr-Tyr^7^-Thr-NH_2_ (D9) and H-D-Tyr^1^-Tyr^2^-Tyr^3^-D-Trp-Lys-Thr-Tyr^7^-Thr-NH_2_ (D91), as the configuration of the amino acid in the first position is the only difference between these two analogs. While the non-optimized logP calculation yields the same result for both, after optimization, L-Tyr seems to increase the lipophilicity of the whole peptide. This is not to say that L- amino acids are more lipophilic in general, as it might be dependent on the neighboring residues as well. What should be noted though is the U-shape that the L-analogs take after optimization, which is visibly different from the straighter D-analog structures.

The effect of tyrosine positioning can be studied when comparing structures of H-L-Tyr^1^-Tyr^2^-Tyr^3^-D-Trp-Lys-Thr-Phe-Thr-NH_2_ (D6) and H-L-Tyr^1^-Tyr^2^-Phe-D-Trp-Lys-Thr-Tyr^7^-Thr-NH_2_ (D7), where three phenylalanine residues have been replaced by tyrosine at different positions. Although they differ in non-optimized structures, when optimized, they curl up into similar shapes and experience the same logP coefficient.

Optimizing all structures results in higher logP values, which means that their hydrophobic parts are more exposed. Overall, the addition of multiple polar residues diminishes the BIM-23052 drug properties, but the addition of a single tyrosine has a desirable result of increased lipophilicity and hence higher membrane permeability.

Finally, docking calculations show that substitution of one Phe with Tyr at position 1 of the DD8 molecule leads to a change in selectivity according to the SSTRs. While the parent compound BIM-23052 demonstrates weak selectivity toward SSTR and bound SSTRs 3, 4 and 5 equally well, the newly synthesized analog DD8 has a narrow selectivity toward SSTR 1 (total energy of the complex is significantly higher than those of the complexes with other receptors).

### 4.3. Hydrolytic Stability

The obtained data in the two model systems used, which mimic human pH in the stomach (pH 2.0) and small intestine (pH 9.0), show complete stability of the newly synthesized compounds at pH 2.0 for 24 h. At pH 9.0, the most active compound DD8 has relatively high stability, and after 24 h only 30% hydrolysis.

## 5. Conclusions

Synthetic SST analogs that have a much longer half-life than the endogenous SST are more useful in the management of acromegaly and numerous neuroendocrine tumors. BIM-23052 is a linear SST analog, which displays large binding affinity to SSTR 3, 4 and 5. Herein, we have investigated the structure–property relationship for a series of BIM-23052 analogs that have undergone structural modifications, and we identified those changes that lead to increased biological activity. The simple strategy used to produce BIM-23052 analogs was to replace Phe residue(s) with Tyr.

Multiple analyses of the obtained data reveal that compound DD8 (D-Phe-Phe-Phe-D-Trp-Lys-Thr-Tyr^7^-Thr-NH_2_), where one Phe is replaced by Tyr, has the best activity against the studied tumor cell lines. The logP values obtained from the in silico calculations show that DD8 is more lipophilic than BIM-23052 and thus is expected to have higher membrane permeability. At the same time, this analog has a narrow selectivity toward SSTR 1. Taking into account that somatostatin analogs bind specific receptors on the surface of tumor cell lines, the obtained data herein show that between all newly synthesized Tyr analogs of BIM-23052, compound DD8 is the best candidate as an anticancer agent for oral administration, which could penetrate the cell membrane and start a different mechanism of tumor cell apoptosis than that characteristic for SST and its analogs. The additional substitution of Phe with Tyr residues does not lead to a better antiproliferative effect, i.e., there is no cumulative effect. However, replacement of the D-amino acid (compound D91) with L-one in the first position (compound D9) seems to slightly increase the lipophilicity of the peptide but does not increase the activity, which indicates that the binding to the SSTRs is the preferable mechanism of action of BIM-23052 Tyr analogs.

## Figures and Tables

**Table 1:** Structures, data and constants of newly synthesized analogs of BIM-23052.

Code	Structure	Molecular Formula	Mw Exact	[M + H]^+^ Found	RT (min)	M.p. (°C)	$\alpha_{546}^{20}$
D11	D-Tyr^1^-Phe-Phe-D-Trp-Lys-Thr-Phe-Thr-NH_2_	C_61_H_75_N_11_O_11_	1137.57	1138.70	3.531	121–123	+8
DD8	D-Phe-Phe-Phe-D-Trp-Lys-Thr-Tyr^7^-Thr-NH_2_	C_61_H_75_N_11_O_11_	1137.57	1138.65	6.845	108–110	−10
D101	D-Phe-Tyr^2^-Tyr^3^-D-Trp-Lys-Thr-Phe-Thr-NH_2_	C_61_H_75_N_11_O_12_	1153.56	1154.60	4.322	118–120	−74
D61	D-Phe-Tyr^2^-Tyr^3^-D-Trp-Lys-Thr-Tyr^7^-Thr-NH_2_	C_61_H_75_N_11_O_13_	1169.55	1170.80	4.009	123–125	+2
D91	D-Tyr^1^-Tyr^2^-Tyr^3^-D-Trp-Lys-Thr-Tyr^7^-Thr-NH_2_	C_61_H_75_N_11_O_14_	1185.55	1186.80	3.420	125–127	−46

**Table 2:** Structures, data and constants of newly synthesized analogs of BIM-23052 containing L-amino acid at position 1.

Code	Structure	Molecular Formula	Mw Exact	[M + H]^+^ Found	RT (min)	M.p. (°C)	$\alpha_{546}^{20}$
D6	L-Tyr^1^-Tyr^2^-Tyr^3^-D-Trp-Lys-Thr-Phe-Thr-NH_2_	C_61_H_75_N_11_O_13_	1169.56	1170.60	3.567	110–112	−20
D7	L-Tyr^1^-Tyr^2^-Phe-D-Trp-Lys-Thr-Tyr^7^-Thr-NH_2_	C_61_H_75_N_11_O_13_	1169.56	1170.65	1.869	81–83	−28
D9	L-Tyr^1^-Tyr^2^-Tyr^3^-D-Trp-Lys-Thr-Tyr^7^-Thr-NH_2_	C_61_H_75_N_11_O_14_	1185.55	1186.65	1.606	78–80	−16

**Table 3:** Cytotoxic and antiproliferative effects of the studied BIM-23052 analogs.

Compounds	Mean IC_50_ ± SD (μm)			
	Cytotoxicity	Antiproliferative Effect		
	BALB 3T3	MCF-10A	MCF-7	MDA-MB-231
BIM-23052	483.10 ± 32.11	203.80 ± 6.32	120.00 ± 6.53	334.80 ± 15.40
D11	1634.40 ± 51.18	864.04 ± 52.27	873.15 ± 26.55	1722.97 ± 58.60
D6	1324.11 ± 53.7	190.98 ± 6.8	358.87 ± 16.13	290.54 ± 12.68
D7	736.91 ± 19.98	432.82 ± 23.15	402.84 ± 24.93	781.99 ± 18.97
DD8	482.93 ± 33.88	161.28 ± 7.97	235.43 ± 14.3	240.89 ± 13.36
D61	1200.52 ± 100.13	554.69 ± 21.28	426.42 ± 23.87	798.18 ± 24.38
D9	1429.3 ± 40.72	394.97 ± 15.29	476.81 ± 31.44	1411.66 ± 19.08
D91	974.67 ± 81.71	540.27 ± 27.21	527.48 ± 54.19	849.45 ± 21.21
D101	1401.35 ± 52.31	604.46 ± 24.66	717.13 ± 20.76	1399.37± 19.98
Cisplatin	537.35 ± 40.27	64.35 ± 2.11	43.07 ± 3.16	6.94 ± 0.47

**Table 4:** Selectivity of the studied BIM-23052 analogs.

Code	SI *	
	MCF-7	MDA-MB-231
BIM-23052	1.7	0.61
D11	0.99	0.50
D6	0.53	0.66
D7	1.07	0.55
DD8	0.69	0.67
D61	1.3	0.69
D9	0.83	0.28
D91	1.02	0.64
D101	0.84	0.43
Cisplatin	1.49	9.27

* Selectivity index, SI = IC_50_ MCF-10A/IC_50_ tumor cell line.

**Table 5:** LogP values of targeted structures.

Code	Structure	LogP	
		Non-Pre-Optimized Structures	Pre-Optimized Structures
BIM-23052	H-D-Phe-Phe-Phe-D-Trp-Lys-Thr-Phe-Thr-NH_2_	0.74	0.83
DD8	H-D-Phe-Phe-Phe-D-Trp-Lys-Thr-Tyr^7^-Thr-NH_2_	0.46	1.17
D101	H-D-Phe-Tyr^2^-Tyr^3^-D-Trp-Lys-Thr-Phe-Thr-NH_2_	0.17	0.88
D61	H-D-Phe-Tyr^2^-Tyr^3^-D-Trp-Lys-Thr-Tyr^7^-Thr-NH_2_	−0.11	0.60
D9	H-L-Tyr^1^-Tyr^2^-Tyr^3^-D-Trp-Lys-Thr-Tyr^7^-Thr-NH_2_	−0.40	0.38
D91	H-D-Tyr^1^-Tyr^2^-Tyr^3^-D-Trp-Lys-Thr-Tyr^7^-Thr-NH_2_	−0.40	0.31
D6	H-L-Tyr^1^-Tyr^2^-Tyr^3^-D-Trp-Lys-Thr-Phe-Thr-NH_2_	−0.11	0.55
D7	H-L-Tyr^1^-Tyr^2^-Phe-D-Trp-Lys-Thr-Tyr^7^-Thr-NH_2_	0.17	0.55
D11	H- D-Tyr^1^-Phe-Phe-D-Trp-Lys-Thr-Phe-Thr-NH_2_	0.46	1.14

**Table 6:** Hydrolytic stability of newly synthesized peptides at pH 9.0 for 24 h.

Code	% Non-Hydrolyzed Product
D6	47
D61	74
D7	54
DD8	72
D9	78
D91	59
D11	61
D101	70

**Table 7:** Calculated total energy of ligand–peptide complexes.

Ligand	Total Energies of the Ligand with the Respective Receptor Type, kJ mol^−1^				
	SSTR1	SSTR2	SSTR3	SSTR4	SSTR5
S1 *	−175.29	−159.55	−322.74	−234.00	−283.73
D6	−114.29	−137.57	−295.45	−251.96	−240.35
DD8	−236.00	−189.25	−152.94	−147.97	−125.25

* Data are already published in [27].

## Data Availability

Small amounts of the target compounds are available from the corresponding author.
